# The Long-Term Effects of Early Childhood Resilience Profiles on School Outcomes among Children in the Child Welfare System

**DOI:** 10.3390/ijerph19105987

**Published:** 2022-05-14

**Authors:** Susan Yoon, Fei Pei, Juan Lorenzo Benavides, Alexa Ploss, Jessica Logan, Sherry Hamby

**Affiliations:** 1 College of Social Work, The Ohio State University, Columbus, OH 43210, USA; benavides.35@buckeyemail.osu.edu (J.L.B.); ploss.4@buckeyemail.osu.edu (A.P.); 2 School of Social Work, Falk College, Syracuse University, Syracuse, NY 13244, USA; fpei01@syr.edu; 3 Quantitative Research, Evaluation and Measurement, College of Education and Human Ecology, The Ohio State University, Columbus, OH 43210, USA; logan.251@osu.edu; 4 Department of Psychology, The University of the South, Sewanee, TN 37383, USA; sherry.hamby@lifepathsresearch.org; 5 Life Paths Research Center, Sewanee, TN 37375, USA

**Keywords:** resilience, school outcomes, academic achievement, school involvement, adverse childhood experiences (ACEs), children, latent profile analysis

## Abstract

This study aimed to examine the association between early childhood resilience profiles and later school outcomes (academic achievement and school involvement) among children in the U.S. child welfare system. This study compared 827 children aged 3–5 years in three latent profile groups (poor emotional and behavioral resilience, low cognitive resilience, and multi-domain resilience) to their baseline profiles using data from the National Survey of Child and Adolescent Well-Being (NSCAW-II). At the three-year follow-up, children with low emotional and behavioral resilience profiles and children with the multi-domain resilience profile had significantly higher basic reading skills, reading comprehension, and math reasoning compared to children with low scores on the cognitive resilience profile. Furthermore, children with the multi-domain resilience profile had significantly higher levels of emotional school engagement than did those with the low emotional and behavioral resilience profile and considerably higher levels of behavioral school engagement compared to those with the low cognitive resilience profile. The findings highlight the persistent effects of early resilience into the later childhood years. Moreover, our results suggest the need for early identification of and intervention for children with low cognitive or emotional/behavioral resilience during the preschool years to promote academic success and school engagement during the school-age years.

## 1. Introduction

Building resilience during early childhood has long-lasting positive effects on an individual’s well-being over the life course. There is a growing body of research on resilience among children who have experienced childhood maltreatment. Recently, an emerging line of research has utilized person-centered approaches (e.g., cluster analysis, latent class analysis, latent profile analysis, or growth mixture modeling) to explore patterns of resilience in populations with a history of childhood trauma and maltreatment. Although recent research has made remarkable headway towards identifying resilience patterns in maltreated children, how these patterns affect later developmental functioning, such as school outcomes, remains unclear and understudied. Examining school outcomes related to early childhood resilience (defined here as a process of achieving positive adaptation across multiple domains of functioning despite exposure to trauma [1] during the preschool years) is crucial in maintaining and strengthening early developmental assets and milestones. This paper builds upon our previous work that identified three distinct profiles of early childhood resilience (i.e., low cognitive resilience, low emotional and behavioral resilience, and multi-domain resilience) among child-welfare-involved children [2]. In this paper, we seek to investigate the extent to which these resilience profiles predict later school outcomes, including academic achievement and school engagement, after three years.

### 1.1. Resilience in Children with a History of Child Maltreatment

Children who have experienced child maltreatment often face adverse psychosocial and behavioral outcomes in later life [3,4]. Specifically, there is a known association between maltreatment and poor social adjustment, juvenile delinquency, psychiatric conditions, low education attainment, and substance abuse [5,6,7]. Several challenges persist for this population as they experience continued exposure to violence, separation from parents/caregivers, multiple out-of-home placements, or re-traumatization [8].

Fortunately, there is a plethora of research on resiliency and protective factors that help support healthy development [9,10]. These studies provide empirical evidence that these children can achieve positive adaptation despite their maltreatment [11,12,13,14,15]. Moreover, a robust body of research has found that certain protective factors help mitigate the harmful effects of childhood maltreatment [16,17]. Among these are personal attributes such as self-efficacy, emotion regulation, temperament, or future orientation; family and cultural supports such as parental emotional support, cognitive stimulation, or parent–child attachment; school/community resources and characteristics such as community cohesion, schools, or peer support [4,16,17,18,19]. Taken cumulatively, these studies recognize the importance of employing a strengths-based perspective to examine resilience and protective factors among children with a history of child maltreatment, as well as moving beyond deficit models that focus on risks and adverse outcomes.

### 1.2. Measuring Resilience and Identifying Patterns of Resilience

Resilience has been historically difficult to measure due to the dynamic nature of the changes and development across the various domains of human function [9]. The literature includes disparate methods and instruments to assess an individual’s resilience and adaptation to hardship, particularly in studies that focus on adults and children with a history of maltreatment [20]. Studies involving children frequently measure functioning across developmental domains (social, cognitive, emotional, behavioral, and occupational) and achievement milestones [9]. In contrast, studies that measure resilience in adults often include the preclusion of psychopathology, measurements of well-being, and social competency [21]. However, studies remain varied throughout both groups.

Although measurement differences remain a challenge in research, multipoint resilience indicators remain viable for comprehensively assessing resilience across different developmental domains [16]. Researchers have argued for considering multiple domains of child functioning when examining resilience among children with a history of maltreatment [9,16,22], as competence and resilience within one domain do not guarantee competence and resilience in another domain. Altogether, child maltreatment and resilience researchers have suggested that resilience measures should be comprehensive and expansive to capture the many facets of resilience and adaptation among children with maltreatment histories.

Recently, an emerging line of research has explored different patterns of resilience, using person-centered approaches rather than variable-centered approaches. While a variable-centered approach focuses on examining the relationship among variables, a person-centered approach identifies heterogeneous subgroups of individuals who share distinct characteristics and attributes [23]. Thus, a person-centered approach is useful in identifying unique and distinct configurations of resilience. One study used a latent profile analysis with a sample of 164 emancipated foster youth and identified four resilience profiles, including maladaptive (16.5%), resilient (47%), internally resilient (30%), and externally resilient (6.5%) [24]. Another study conducted a latent profile analysis with 12-year-old children who had been involved with child protective services (CPS). The study identified five profiles of adaptation/competence, including consistent resilience (12.7%), consistent maladaptation (11.6%), posttraumatic stress problems (8.9%), school maladaptation/family protection (36.2%), and low socialization skills (30.6%) [25]. Other studies, not specifically focused on child maltreatment, explored resilience patterns among children who have experienced early adversities and trauma (such as exposure to intimate partner violence, poverty, or parental psychopathology), and these studies likewise identified four or five patterns of resilience [15,26].

### 1.3. Early Childhood Resilience Profiles and Later School Outcomes

**Figure 1:**
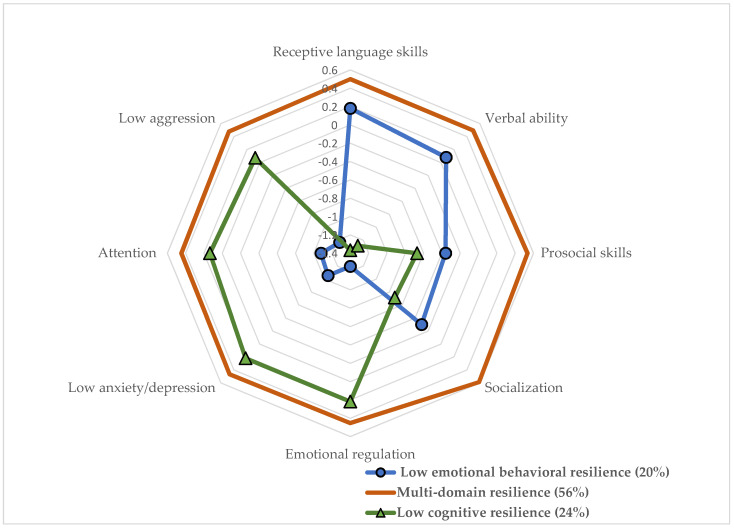
Radar Chart of Early Childhood Resilience Profiles. Note. Adapted from [2]. z-scores (*M* = 0, *SD* = 1) of the resilience indicators were used for the radar chart for interpretability purposes.

Our team’s prior work focused on the identification of profiles of resilience, specifically during early childhood (the ages of 3 to 5 years) among child-welfare-involved children as this important developmental period remained overlooked in the maltreatment and resilience profile literature [2]. Using a latent profile analysis, we identified three distinct profiles of resilience: low emotional and behavioral resilience (20%), low cognitive resilience (24%), and multi-domain resilience (56%). The low emotional and behavioral resilience profile had children who showed the lowest emotional and behavioral adaptations yet above-average levels of cognitive and social functioning. The low cognitive resilience profile included children who showed the lowest levels of cognitive ability and lower levels of social functioning, yet average levels of emotional and behavioral functioning. Finally, the multi-domain resilience profile included children who demonstrated above-average levels of competence across all domains of functioning (Figure 1 provides a visual representation of the three latent profiles).

Despite emerging research exploring distinct configurations of resilience among children with maltreatment histories, including our work of three early childhood resilience profiles, little research has examined how resilience profiles in early childhood are associated with distal outcomes such as school outcomes at a later developmental stage. School context and outcomes are critical to examine as they have important short- and long-term implications for elements of quality of life, including child self-esteem/self-worth, mental and behavioral well-being, and later employment status and job satisfaction during adulthood [27,28,29,30,31]. For example, school engagement is a protective factor for maltreated children [32]. Specifically, researchers have found that children with adverse childhood experiences demonstrate high levels of well-being, including higher self-esteem, when they have high levels of high school engagement [33,34]. Youth acquire a sense of belongingness and purpose when they feel connected to their school and the staff [35,36]. Unfortunately, however, research has also shown that although school is an important change agent, children with a history of maltreatment are likely to quickly disengage from school for reasons such as placement or school instability and peer victimization [35]. Similarly, children with maltreatment histories often experience academic difficulties and poorer academic achievement, including lower scores on standardized reading and math tests [37,38].

To date, most studies have examined resilience profiles as an outcome and have focused on identifying predictors or characteristics associated with resilience profiles [15,25,26]. Consequently, there is a dearth of research on the relationship between early childhood resilience profiles and distal outcomes such as later school outcomes (e.g., academic achievement or school engagement) among children involved with the child welfare system. Understanding how early childhood resilience affects later development and outcomes is vital to developing intervention strategies that will maximize the likelihood of ongoing, uninterrupted resilient development. Although not directly focused on children with maltreatment histories, one study found that children who grew up in poverty but achieved a high threshold of resilience by the time they entered kindergarten showed academic achievement during the elementary school years comparable to that of children not in poverty [39], providing preliminary evidence that resilience in early childhood predicts later school success.

### 1.4. The Current Study

Building upon our prior work on early childhood resilience profiles [2], we examined the resilience profiles’ long-term relations to academic achievement and school engagement during the school-age years. Understanding the long-term influence of early childhood resilience on school outcomes is vital to providing effective support and interventions that foster continued resilient development, including success in school, for this vulnerable population. The following research question guided the study: how are different profiles of resilience at baseline associated with academic achievement (e.g., basic reading skills, reading comprehension, or math reasoning) and school engagement (emotional or behavioral) when measured after three years? It was hypothesized that children with the multi-domain resilience profile at baseline would have greater academic achievement and school engagement compared to those with the low cognitive resilience profile and those with the low emotional and behavioral resilience profile at baseline. 

## 2. Materials and Methods

### 2.1. Data and Sample

This secondary data analysis used the data from the National Survey of Child and Adolescent Well-Being [40], which collected longitudinal data from children and families involved in the child protective services system. From 2008 to 2021, NSCAW-II collected three waves of data among 5872 children up to the age of 17.5: Wave 1 was the baseline, Wave 2 was a 1.5-year follow-up, and Wave 3 was a 3-year follow-up. Since we sought to investigate how early childhood resilience was associated with later school outcomes, the present study analyzed data from Waves 1 and 3. Our analytic sample included children who were 3–5 years old at Wave 1 (N = 827).

Descriptive statistics of all study variables are shown in Table 1. In terms of sample characteristics, children’s mean age in Wave 1 was 3.96 (*SD* = 0.82). Just under half of the children (46.1%) were girls; 39.6% were non-Hispanic White, 31.4% were non-Hispanic Black, 24.0% were Hispanic, and 5.0% were other races, including American Indian, Asian, Native Hawaiian/Pacific Islander, and multiple races. Regarding caregivers’ characteristics, 25.8% of primary caregivers had not completed high school, and about 78.1% reported household income below the 200% federal poverty line.

### 2.2. Measures

#### 2.2.1. Resilience

Resilience was conceptualized as a multidimensional construct and measured using multiple instruments in Wave 1 (baseline). Specifically, cognitive resilience, social resilience, emotional resilience, and behavioral resilience were assessed and operationalized as competence and positive functioning in the domains of cognitive, social, emotional, and behavioral development, respectively, after exposure to adversity [4,41]. Receptive language skills and verbal ability (expressive language skills) in the domain of cognitive resilience were measured using two standardized, validated scales, the Expressive Communication subscale and the Auditory Comprehension subscale, within the Preschool Language Scale—3 [42]. PLS-3, which showed strong validity [43], is a standardized tool to assess children’s overall language development from birth to age 6. The standard sum scores were calculated for these two subscales, with higher scores indicating better receptive language skills and greater verbal ability. Internal consistency of these two subscales was good at α = 0.87 for the Expressive Communication scale and α = 0.85 for the Auditory Comprehension scale. The domain of social resilience was measured using two scales: the 39-item Social Skills Rating System [44] and the 15-item Vineland Adaptive Behavior Scale Screener [45]. The SSRS assessed children’s prosocial skills, and the VABSS measured children’s functioning in social situations (socialization). Standard scores were calculated for both the SSRS and the VABSS, with higher scores indicating higher levels of social functioning. Internal consistencies of the two scales were acceptable in this sample (SSRS α = 0.91; VABSS α = 0.75). For the domain of emotional resilience, emotional regulation and anxiety/depression were assessed using two subscales of the Child Behavior Checklist for Ages 1.5–5 (CBCL/1.5–5; Achenbach and Ruffle, 2000): the Emotionally Reactive scale (8 items; α = 0.78) and the Anxious/Depressed scale (8 items; α = 0.63), respectively. Finally, for the domain of behavioral resilience, child aggression and attention were measured using the 8-item CBCL Aggression scale (α = 0.82) and the 8-item CBCL Attention Problem scale (α = 0.91).

#### 2.2.2. Academic Achievement

At the 3-year follow-up, the Woodcock–Johnson III Tests of Achievement (WJ-III; Woodcock et al., 2003) was employed to evaluate children’s academic achievement. Three subscales of WJ-III, the Letter–Word Identification scale, the Passage Comprehension scale, and the Applied Problems scale, were used to capture children’s basic reading skills, reading comprehension, and math reasoning, respectively. For each subscale, the standard sum scores of the items were used, with higher scores representing better achievement/performance.

#### 2.2.3. School Engagement

At the 3-year follow-up, children’s school engagement was evaluated using the Drug-Free Schools Outcome Study Questions (DFSCA). This scale has been widely used to measure school engagement among children involved in the child welfare system (α = 0.72). Two aspects of school engagement were assessed based on a study that found the two-factor structure of the scale to fit the data well [46]. Specifically, the scale measured emotional engagement (3 items, such as “enjoy being in school” and “find classes interesting”) and behavioral engagement (6 items, such as “do best work in school,” “get along with other students,” and “get sent to the office because misbehaved”). The youth’s responses to the items were summed (1 = never, 2 = sometimes, 3 = often, 4 = almost always) to create emotional engagement and behavioral engagement scores. Negatively worded items were reverse-coded, so higher scores meant better school engagement.

#### 2.2.4. Covariates

Demographic variables, including children’s age, sex (0 = male; 1 = female), and race/ethnicity, were controlled for in the present study. Child race/ethnicity was a categorical variable and was dummy coded into White, Hispanic, and Other, using Black as a reference group. Other information was added as covariates, including child abuse (0 = no exposure to child abuse; 1 = at least one CPS report for alleged child abuse), neglect (0 = no exposure to child neglect; 1 = at least one alleged incident of child neglect), poverty (0 = household income ≥ federal poverty level; 1 = household income < federal poverty level), and caregiver’s highest education level (0 = high school or more; 1 = less than high school).

### 2.3. Data Analysis

A latent profile analysis (LPA) was previously conducted to examine heterogeneity in resilience among children involved with the child welfare system and revealed three distinct resilience profiles (i.e., low emotional and behavioral resilience, low cognitive resilience, and multi-domain resilience) [2]. Building upon these findings, we performed a three-step LPA to examine the relationship between the three resilience profiles and the 3-year follow-up school outcomes (distal outcomes). The three-step LPA method is generally considered more robust and rigorous than the one-step LPA approach [47,48,49,50]. In the first step, unconditional LPA models were estimated to determine the optimal number of classes (this step overlaps with the previous analysis whereby we identified three resilience profiles) [2]. The second step involved assigning each child to a latent class based on the posterior probability obtained in the first step. Finally, in the third step, the conditional LPA model examined the mean differences in the five distal outcomes (three academic achievement outcomes and two school engagement outcomes) across the identified resilience profiles while controlling covariates. Missing data were handled by the full maximum likelihood method. SPSS Version 27.0 [51] was used for data management and descriptive analysis, and Mplus Version 8.0 [23] was used to conduct the three-step LPA.

## 3. Results

To address our research question, we examined how the three resilience profiles at baseline—low emotional and behavioral resilience (20%), low cognitive resilience (24%), and multi-domain resilience (56%) [2] were associated with children’s academic achievement and school engagement measured three years after. Table 2 shows the results of pairwise mean comparisons for the five school outcomes across the three resilience profiles. Children with the multi-domain resilience profile showed significantly higher levels of basic reading skills (mean difference = 13.18, *p* < 0.001), reading comprehension (mean difference = 12.60, *p* < 0.001), and math reasoning (mean difference = 14.82, *p* < 0.001), compared to children with the low cognitive resilience profile. Similarly, children with the low emotional and behavioral resilience profile showed significantly higher levels of basic reading skills (mean difference = 10.81, *p* < 0.01), reading comprehension (mean difference = 10.13, *p* < 0.001), and math reasoning (mean difference = 12.16, *p* < 0.001), compared to children with the low cognitive resilience profile. There were no significant mean differences in academic achievement between children with the multi-domain resilience profile and children with the low emotional and behavioral resilience profile.

Regarding school engagement, children with the multi-domain resilience profile at baseline showed significantly higher levels of emotional engagement (mean difference = 0.80, *p* = 0.041) compared to children with the low emotional and behavioral resilience profile. Additionally, children with the multi-domain resilience profile at baseline showed significantly higher levels of behavioral school engagement (mean difference = 1.17, *p* = 0.033) compared to children with the low cognitive resilience profile. There were no other significant mean differences in school engagement among the three resilience profiles. The effects of covariates on academic achievement and school engagement distal outcomes are shown in Table 3. Girls had showed significantly higher levels of basic reading skills, reading comprehension, and behavioral engagement compared to boys. Older age at baseline was associated with lower levels of basic reading skills and reading comprehension but with higher levers of behavioral engagement during the school-age years. Poverty, child abuse, and child neglect were all significantly associated with lower levels of basic reading skills and reading comprehension during the school-age years.

## 4. Discussion

The primary aim of this study was to examine the extent to which unique profiles of resilience (low emotional and behavioral resilience, low cognitive resilience, multi-domain resilience) in early childhood predict future school outcomes, including academic achievement and school engagement, three years later, in children involved with the child welfare system. The study findings provide new insight into the long-term effects of early childhood resilience on later development among children involved with the child welfare system.

Our hypothesis that children with the multi-domain resilience profile would have greater academic achievement compared to those with the low cognitive or emotional/behavioral resilience profile was partially supported. Children with the multi-domain resilience profile had greater academic achievement compared to those with the low cognitive resilience profile but did not do any better or worse than those with the low emotional and behavioral resilience profile. Interestingly, not just children with the multi-domain resilience profile, but also children with the low emotional and behavioral resilience profile showed significantly higher levels of basic reading skills, reading comprehension, and math reasoning compared to those who had the low cognitive resilience profile at baseline. In other words, children who showed low cognitive competence during early childhood continued to show those lower skills as they entered school. These findings corroborate the broader literature that documents a link between early cognitive skills and later academic performance among high-risk children, including children in low-income families [52] and children with a history of child maltreatment [53]. Collectively, our findings may suggest that these children could benefit from more targeted support and highlight the potential importance of early intervention to support the successful development of cognitive skills during early childhood.

Overall, resilience profiles during the preschool years were not a strong predictor of the level of school engagement at the three-year follow-up. It should be noted that there is still some ambiguity about the factor structure of the school engagement measure (the Drug-Free Schools and Communities Act scale) used in this study [46]. Some studies have used it as a unidimensional scale, whereas others have argued for the two-factor (emotional and behavioral) or the three-factor (emotional, behavioral, and cognitive–behavioral) structure [54,55,56,57]. We opted to use the two-factor structure to assess emotional engagement and behavioral engagement in school because this structure has consistently shown robust evidence for construct validity in prior research [46], but it is possible that other factor structures might have fit the data better.

Nonetheless, there were two significant findings for school engagement. First, children with the multi-domain resilience profile showed significantly higher levels of emotional school engagement compared to those with the low emotional and behavioral resilience profile. Our first finding is partially consistent with prior studies that measured emotional school engagement and other areas associated with child internalizing and externalizing behaviors [58,59,60]. These studies found anger, measured in preschool [58] and kindergarten [60], to be negatively associated with emotional and behavioral engagement in kindergarten. Although it is somewhat surprising and inconsistent with prior research that children exhibiting low emotional engagement would still be behaviorally engaged and doing well academically, these findings are consistent in part with the study of Bryce et al. (2018) in which academic achievement was positively associated with behavioral engagement, but not emotional engagement. Our finding suggest that the strong cognitive skills among the children with the low emotional and behavioral resilience profile may have allowed them to participate and do well academically, despite negative feelings towards school. While these children are behaviorally engaged and doing well academically, it is possible that emotional engagement among children in the low emotional and behavioral resilience profile may be hindered by poor relationships with peers and/or teachers. While research specifically measuring emotional engagement among early school-age children remains relatively limited, existent literature has found positive peer relationships to be connected to high emotional engagement [61,62]. Conversely, lower emotional engagement has been connected to higher teacher–student and peer conflict [62,63]. Children lower in emotional and behavioral functioning may struggle to make positive relational connections, diminishing their sense of belonging and emotional connectedness to school.

Our second significant finding related to school engagement revealed that children with the multi-domain resilience profile showed significantly higher levels of behavioral school engagement than those with the low cognitive resilience profile did. This finding is aligned in part with previous literature that has found that language and behavior problems often co-occur [64] and that children with language delays are more likely to develop later behavior problems [65]. Considering children with low cognitive resilience profiles displayed average levels of behavioral functioning during the preschool period, later issues with behavioral school engagement during the school-age years may be connected to their lower cognitive skills. As evidenced by their academic performance, children with the low cognitive resilience profile displayed lower levels of basic reading skills and reading comprehension three years later. This supports elements of Stanovich’s (1986) Matthew effects model, in which difficulty with reading early on “results in unrewarding early reading experiences that lead to less involvement in reading-related activities” (p. 364). Our findings are consistent with other research that found that, after controlling for prior behavior and attention problems as well other SES and demographic confounds, poor reading ability in first grade significantly predicted children’s behavior in third grade, as these children were more likely to be task avoidant, act out, withdraw from classroom activities, and display poor self-control [66].

Given their struggles in academic performance and behavioral engagement, it is somewhat surprising that children with the low cognitive resilience profile did not display significantly lower emotional engagement, especially considering they displayed lower social functioning at baseline. This could be explained by the significant change in demands from preschool to school age [67]. While lower receptive and expressive language skills during preschool age may have inhibited social functioning, they likely had little impact on behavioral functioning, given that the behavioral demands at preschool age are relatively low. However, once children entered school age, their lower cognitive skills may have affected their ability to meet increased academic needs, leading to disengagement. At the same time, social demands may have not been as challenging to achieve, likely allowing children to meet these demands despite their lower cognitive skills.

### 4.1. Limitations

Like other empirical studies, this study is not without its limitations. First, the use of a sample comprising children involved with the child welfare system due to child maltreatment may limit the generalizability of the study results. Second, despite the temporal order of measuring resilience at baseline and school outcomes 3 years later, any firm causal inference may not be made, because other, unobserved factors may play a role in the associations between these variables. Third, although it is a strength that we utilized multiple indicators and measures of resilience to identify resilience profiles, our measures of cognitive resilience were heavily focused on language ability and did not assess other aspects of cognitive ability, such as reasoning, remembering, problem solving, or decision making. Future research may benefit from including more comprehensive measures of cognitive resilience.

### 4.2. Implications

The study’s findings offer important insights into practice and policy development to promote the educational well-being of children with a history of CPS involvement. Considering that our results indicated less optimal school outcomes among children who had low cognitive resilience or low emotional and behavioral resilience during early child-hood, it is important to target these children for early identification and intervention to ensure their academic success and school engagement during the school-age years. For instance, it is crucial to test whether interventions, such as Dialogic Reading [68], designed to improve language ability among young children with low cognitive resilience (i.e., those experiencing challenges with language development) provide long-term benefits for children’s academic achievement and behavioral engagement in school during the school-age period. Similarly, given the lower levels of emotional school engagement among children who had low emotional and behavioral resilience during early childhood, targeted interventions, such as trauma-focused cognitive behavioral therapy (TF-CBF) [69], may be helpful in addressing emotional and behavior problems. Further, it may be particularly important to implement interventions during early school age that help children gain a positive image, thoughts, and emotions about school, especially considering the decline in school engagement trajectories among many students as they progress through elementary school [63,70]. Utilizing school-based interventions that use integrative strategies to target the various dimensions of school engagement may be most appropriate. Such integration may increase effectiveness while also addressing a wide variety of different students’ engagement needs. Considering that children in this study exhibited generally moderate levels of emotional and behavioral school engagement regardless of their early resilience profiles, it seems essential to devise more effective strategies to boost school interest and engagement among children involved with the child welfare system.

## 5. Conclusions

We examined the longitudinal associations between early childhood resilience profiles (low emotional and behavioral resilience, low cognitive resilience, multi-domain resilience) and school outcomes (academic achievement; emotional and behavioral school engagement) among children involved with the U.S. child welfare system. Overall, the study results indicated the lasting effects of early resilience into the later childhood years, demonstrating the virtuous cycles of resilience. Compared to children with the multi-domain resilience profile, children with the low emotional and behavioral resilience profile had comparable academic achievement and were behaviorally engaged in school but displayed lower emotional engagement. In contrast, children with the low cognitive resilience profiles had poorer academic achievement and were not behaviorally engaged, yet still displayed high levels of emotional engagement. Collectively, our findings may suggest that while academic achievement and behavioral engagement may be closely connected, emotional engagement may depend on other factors outside of how well a child is doing in school or how behaviorally engaged they are. It should be noted that multiple mechanisms that were not examined in the study, such as social ecological strengths and resources available during the preschool years, may have contributed to better school outcomes [71]. Future research is needed to further determine the factors that influence emotional school engagement among early school-age children, the extent to which interpersonal relationships, such as teacher and peer relationships, influence such engagement, and whether such connections are unique to this age group. Further illumination in these areas will allow for more targeted and effective interventions to bolster school engagement and academic achievement among children involved with the child welfare system as they progress throughout their academic careers. Finally, it might be interesting to investigate whether the enduring effects of early childhood resilience observed during the school-age period persist throughout later developmental stages, such as adolescence and young adulthood.

## Figures and Tables

**Table 1:** Descriptive Statistics for Key Study Variables (N = 827).

	%	*M* (*SD*)
Age at baseline (in years)		3.96 (0.82)
Sex (girls)	46.1%	
Race/Ethnicity		
White; Non-Hispanic	39.6%	
Black; Non-Hispanic	31.4%	
Hispanic	24.0%	
Other	5.0%	
Caregiver’s education (less than high school)	25.8%	
Household income ≤ 200% poverty level	78.1%	
Academic Performance		
Letter–Word Identification standard score		98.81 (16.22)
Passage Comprehension standard score		91.21 (14.54)
Applied Problems standard score		97.00 (15.36)
School Engagement		
Emotional engagement		9.04 (2.29)
Behavioral engagement		20.25 (3.23)

Note. Adapted from [2]. Other race included American Indian, Asian, Native Hawaiian/Pacific Islander, and Multiple race categories.

**Table 2:** Distal Mean Differences between Four Latent Classes.

School Distal Outcome	Class	Distal Mean	Low Cognitive Resilience	Multi-Domain Resilience
Letter–Word Identification	Low emotional, behavioral resilience	121.68	10.81 ***	−2.37
	Low cognitive resilience	110.87	-	−13.18 ***
	Multi-domain resilience	124.06	-	-
Passage Comprehension	Low emotional, behavioral resilience	104.77	10.13 ***	−2.47
	Low cognitive resilience	94.64	-	−12.60 ***
	Multi-domain resilience	107.23	-	-
Applied Problems	Low emotional, behavioral resilience	102.60	12.16 ***	−2.66
	Low cognitive resilience	90.44	-	−14.82 ***
	Multi-domain resilience	105.26	-	-
Emotional Engagement	Low emotional, behavioral resilience	7.07	−6.66	−0.80 *
	Low cognitive resilience	7.74	-	−0.13
	Multi-domain resilience	7.87	-	-
Behavioral Engagement	Low emotional, behavioral resilience	16.28	0.40	−0.77
	Low cognitive resilience	15.88	-	−1.17 *
	Multi-domain resilience	17.05	-	-

Note. * *p* < 0.05, *** *p* < 0.001.

**Table 3:** The Effects of the Covariates on the Distal Outcomes.

	Letter–Word Identification		Passage Comprehension		Applied Problems		Emotional Engagement		Behavioral Engagement	
	Estimate	S.E.	Estimate	S.E.	Estimate	S.E.	Estimate	S.E.	Estimate	S.E.
Child sex (girls)	0.43 **	0.13	0.38 **	0.12	0.16	0.12	0.31	0.23	0.88 **	0.31
Child age at baseline	−0.56 ***	0.09	−0.32 ***	0.09	−0.16	0.09	0.25	0.15	0.43 *	0.21
Child race/ethnicity										
White/Non-Hispanic	−0.11	0.16	−0.14	0.14	0.01	0.14	−0.14	0.27	0.42	0.37
Hispanic	−0.20	0.18	−0.24	0.18	0.00	0.17	0.23	0.29	0.46	0.42
Other ^a^	−0.23	0.26	−0.02	0.23	−0.11	0.23	−0.24	0.40	0.69	0.58
Parent education ^b^	0.19	0.15	0.16	0.14	0.26	0.14	−0.05	0.24	0.54	0.36
Poverty ^c^	−0.28 *	0.14	−0.26 *	0.12	−0.19	0.13	0.09	0.23	−0.20	0.3
Child neglect	−0.33 *	0.14	−0.28 *	0.12	0.02	0.13	−0.16	0.23	−0.20	0.32
Child abuse	−0.42 **	0.15	−0.40 **	0.13	−0.28 *	0.13	−0.18	0.23	−0.23	0.34

Note. * *p* < 0.05, ** *p* < 0.01, *** *p* < 0.001; ^a^ other race included American Indian, Asian, Native Hawaiian/Pacific Islander, and Multiple race categories; ^b^ parental education more than high school; ^c^ household income below the federal poverty level.

## Data Availability

This document includes data from the National Survey of Child and Adolescent Well-Being that was developed under contract with the Administration on Children, Youth, and Families, U.S. Department of Health and Human Services (ACYF/DHHS). Restrictions apply to the availability of these data. The data were provided by the National Data Archive on Child Abuse and Neglect (NDACAN).
